# Quantifying robustness against sharp bending in an integrated topological interface of valley photonic crystals

**DOI:** 10.1515/nanoph-2023-0727

**Published:** 2024-02-15

**Authors:** Chao-Heng Guo, Guo-Jing Tang, Meng-Yu Li, Wen-Jie Chen, Xin-Tao He, Jian-Wen Dong

**Affiliations:** School of Physics & State Key Laboratory of Optoelectronic Materials and Technologies, Sun Yat-sen University, Guangzhou 510275, China

**Keywords:** topological photonics, valley photonic crystals, robust propagation, bending loss

## Abstract

Sharp-bending waveguide is a key element for ultra-compact and densely integrated photonic devices, which is promising to enlarge the capability of modern information processing in a single chip. Topological photonics manifest the nature of robust propagation against sharp bending and such robustness has been experimentally demonstrated in topological integrated interfaces. It is important to quantify the bending loss of topological interface but has remained exclusive. In this work, we report on the characterization of sharp-bending robustness in the integrated topological interface of valley photonic crystals (VPCs) by experimentally quantifying the ultralow bending loss. The VPCs are designed on a standard silicon-on-insulator platform with the inversion-symmetry broken in honeycomb lattice, and four types of topological interfaces can be constructed by two topologically-distinct VPCs. As one of the representative cases, zigzag-AA interface is applied to demonstrate the robust propagation along sharp bending. In experiment, we fabricate a series of VPC interfaces with different turn number and the same transmission distance, which perform the ultralow bending loss less than 0.02 dB per 120-deg turning. Furthermore, we experimentally characterize the propagation loss in the integrated interfaces. Our approach not only shows the ability of VPC topological interfaces to suppress backscattering stemming from sharp bending, but also paves the way for topological nanophotonic dense integration.

## 1 Introduction

Photonic integrated circuits (PICs) have made extraordinary progress for modern information processing and one of the goals is the highly dense integration on a single chip to enlarge the processing capability of light [1], [2], [3], [4]. This demand requires the miniaturization of devices such that some of elements should be constructed by sharp-bending waveguides [5], [6], [7]. Conventionally, sharp bent can be achieved by turning the strip waveguide as a certain radius to guarantee the condition of total internal reflection (TIR) [8], [9], which have reduced the bending loss to be less than 0.5 dB/turn. But the nature of TIR makes trade-off between bending loss and turning radius. In other word, one should increase the size of bending waveguides to reduce bending loss, or prefer to compact size regardless of the radiation loss of bending. To overcome this issue, variety of new strategies have been proposed with relating to metamaterials [6], [7], [10], [11], [12], [13], [14] such as the line-defect photonic crystal slabs that precisely optimize the building blocks at the corners [11] and the e-skid waveguides that clad high-index contrast grating structures to control the skin-depth of evanescent waves [7]. The e-skid waveguides have achieved low bending loss as 0.06 dB/turn with 90-deg bending. Recently, the advances of topological photonics have received significant attentions to control the flow of light in an innovative way [15], [16], [17]. The topological origin of photonic states, such as quantum Hall [18], quantum spin Hall [19], [20], [21] and quantum valley Hall effects [22], [23], [24], maintain the robustness of optical modes propagating along sharp-bending geometry and thus many promote experiments have been demonstrated on integrated topological interfaces [25], [26], [27], [28]. Topological photonics provide a platform to address the demand for high-performance densely integrated optical components.

Valley photonic crystal (VPC), the analogous of quantum valley Hall effect in photonic crystal system, is one of the representative structures in topological photonics [22], [23], [24], [29], [30], [31], [32]. The design of VPC should break the spatial inversion symmetry of photonic crystal slab which is easy to access with all dielectric materials, so that VPC is compatible with PIC platform to develop integrated nanophotonic devices [27], [28], [33], [34], [35], [36], [37], [38]. In principle, the edge states along the interface between two domains of topologically-distinct VPCs are protected against any perturbation that does not mix the two valleys. As a typical instance, the interface of 120-degree-bending can support the high-efficiency turning of light propagation, and thus a large number of experiments from microwave band [39], [40] to near-infrared (NIR) wavelength [27], [28], [41], [42] have successfully verified the robustness of sharp bending. But most of those works are limited on the phenomenological verifications by going through the comparable transmission spectra between sharp-bending and flat interfaces. Bending loss is a key parameter to determine the performance of sharp-bending waveguides. In principle, the bending loss of topological waveguides (e.g. VPC interfaces) should be ultralow, but it is lack of discussion due to the practical challenges in sample fabrication and optical measurement. The measurement of loss index will further promote the application of topological photonic structures in highly integrated devices and systems.

In this work, to characterize the sharp-bending robustness of VPCs, we will show the experimental quantification of the ultralow bending loss along valley-dependent integrated topological interfaces. At first, we prepare the VPCs on 220-nm-thickness silicon layers under the substrate of silica. Derived from a honeycomb-lattice photonic crystal with triangular-profile air holes, the unit cell of such VPC contains two nonequivalent air holes in order to break the inversion symmetry and achieve valley-contrasting topological phase. Second, there are four types of VPC interfaces constructed by two topologically-distinct VPCs, and we introduce the representative case of zigzag-AA interface to demonstrate the robust propagation against sharp bent. Third, we fabricate the samples of the zigzag-AA topological interfaces with different turn number and the same transmission distance. The measured results show that the bending loss is less than 0.06 dB per 120-deg turning, which is an ultralow value in contrast to strip waveguides of PICs. For example, the bending loss can be as low as 0.0143 dB/turn at the wavelength of 1550 nm. Finally, we also perform the measurement of the propagation loss in experiment, which is greater than 10 dB/mm. Our experiments not only show the ability of the VPC topological interface to suppress backscattering caused by sharp bending, but also the qualitative measurement of VPC paves the way for the dense integration of topological nanophotonics.

## 2 Results and discussion

### 2.1 Topological edge states for four types of VPC interfaces

**Figure 1:**
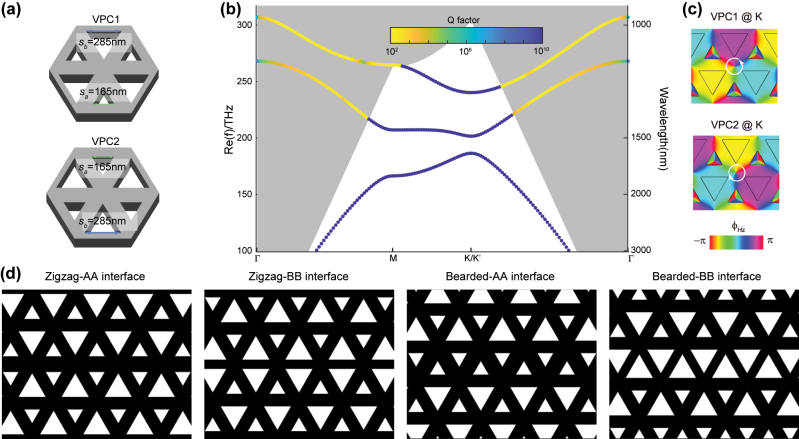
Design of valley photonic crystals (VPCs) and four types of VPC interfaces. (a) Schematic view of VPC1 and VPC2 slabs prepared on a 220-nm-thickness silicon layer. The unit cell of such VPC contains two nonequivalent air holes with triangular geometry. The smaller (bigger) one has side length of *s*_*a*_ = 165 nm (*s*_*b*_ = 285 nm). (b) TE-like bulk bands of VPC1 and VPC2. Between the first and second bands, a TE-like bandgap from 1496 nm to 1610 nm is separated by two band extrema at *K*/*K*′ valleys. The color represents the Q factor of photonic crystal (PC) slab modes and grey region is the light cone of silica. (c) Simulated phase vortex of *H*_*z*_ field profile at *K* valley of the first band. Around the center of a unit cell, the phase vortex of VPC1 at *K* valley increases counterclockwise by 2π, while VPC2 has clockwise phase vortex at the same valley. (d) Four types of topological interfaces formed by two domains of VPC1 and VPC2. Valley physics guarantee the topological features of edge states only projected to Γ*K* direction, which contains two types of stacking (i.e. zigzag and bearded). As the two air holes of unit cell are nonequivalent from spatial inversion-symmetry breaking, the zigzag or bearded case can be stacked by A–A holes or B–B holes.

The VPCs of this work are prepared on silicon-on-insulator (SOI) wafer with 220-nm-thickness silicon layers, which are asymmetrically cladded by the top air region and the bottom silica substrate. As show in Figure 1(a), two types of VPCs (VPC1 and VPC2) with reverse topological invariants are designed in order to achieve valley-dependent edge states. The unit cell of such VPC contains two nonequivalent air holes with triangular geometry, and are periodically arranged in honeycomb lattice with lattice constant of *a* = 454 nm. The smaller (bigger) one has side length of *s*_*a*_ = 165 nm (*s*_*b*_ = 285 nm). Note that both VPC1 and VPC2 are the inversion-symmetry partner with each other, and thus have the same bulk band structures. Figure 1(b) gives the transverse-electric-like (TE-like) bulk bands, calculated by guided-mode expansion method [43]. Between the first and second bands, a TE-like bandgap from 1496 nm to 1610 nm is separated by two band extrema at *K*/*K*′ point, i.e. photonic valleys. The color represents the Q factor of photonic crystal (PC) slab modes and grey region is the light cone of silica. Below light cone, the PC slab modes are regular bound states with infinite Q, such that the out-of-plane radiation can be suppressed in principle.

We have demonstrated the identical bulk band structures of VPC1 and VPC2, but it should be noted that their topological properties are distinct and should be characterized by inequivalent topological invariants. Based on valley-contrasting physics [44], the bulk modes at the two inequivalent *K*/*K*′ valleys are a pair of pseudospins, and thus they carry valley-dependent orbital magnetic momentum

(1) $$\upsilon_{l}\left(K,K^{\prime }\right)=\tau_{z}\mu_{B}^{*},$$

where $\mu_{B}^{*}$ is the effective Bohr magneton at the bottom band. *τ*_*z*_ is a scalar quantity labelled the two valleys, where *τ*_*z*_ = +1 denotes for the *K* valley and *τ*_*z*_ = −1 for the *K*′ valley. For the valley-dependent topological phase, we just need to have the sign of effective Bohr magneton [i.e. $\mathrm{sgn}\left(\mu_{B}^{*}\right)$ ], instead of the exact value of $\mu_{B}^{*}$ . Derived from the topological physics [44], we can have the definition of valley-dependent topological index as

(2) $$C_{K/K^{\prime }}=\tau_{z}\mathrm{sgn}\left(\mu_{B}^{*}\right)/2.$$

In a PC, the effective ‘magnetic’ momentum is related to the phase vortex of electromagnetic fields, where $\tau_{z}\mathrm{sgn}\left(\mu_{B}^{*}\right)=$ +1 or −1 represents that the phase vortex increases clockwise (CW) or counterclockwise (CCW) by 2π. Therefore, it is easy to determine the valley indices by exploring the phase vortex of bulk modes at different valleys. Figure 1(c) shows the *H*_*z*_ phase distributions for the first TE-like modes at the *K* valley. For VPC1, the CCW phase vortex at the *K* valley indicates the topological valley index *C*_K_ = −1/2 and the index for *K*′ valley carry the reverse sign. As a consequence, we have the valley Chern number $C_{\text{V}}=C_{\text{K}}-C_{{\text{K}}^{\prime }}=-1$ for VPC1. It also leads to valley-chirality locking feature that the bulk mode at *K*/*K*′ valley can be selectively excited by using a chiral source with the same phase vortex. On the contrary, the chirality of phase vortex, valley indices and valley Chern number of VPC2 are opposite to those of VPC1.

Due to bulk-edge correspondence, an interface formed by two domains of topologically-distinct VPCs (i.e. VPC1 and VPC2) will support robust propagation of topological edge states. Note that the valley physics guarantee the topological features of edge states only projected to Γ*K* direction, which contains two types of stacking (i.e. zigzag and bearded). On the other hand, as the two air holes of unit cell are nonequivalent from spatial inversion-symmetry breaking, the zigzag or bearded case can be stacked by A–A holes or B–B holes. In all, there are four types of topological interfaces formed by two domains of VPC1 and VPC2, as schematically shown in Figure 1(d). Note that these two types of stacking cases have different symmetries. The zigzag-stack configuration is mirror symmetry with respect to *y* axis, while the bearded configuration possesses glide-plane symmetry, such that the interface is invariant under a half-lattice-constant translation along *x* axis and a mirror operation along *y* axis.

### 2.2 Optical properties of edge states along zigzag-AA topological interface

**Figure 2:**
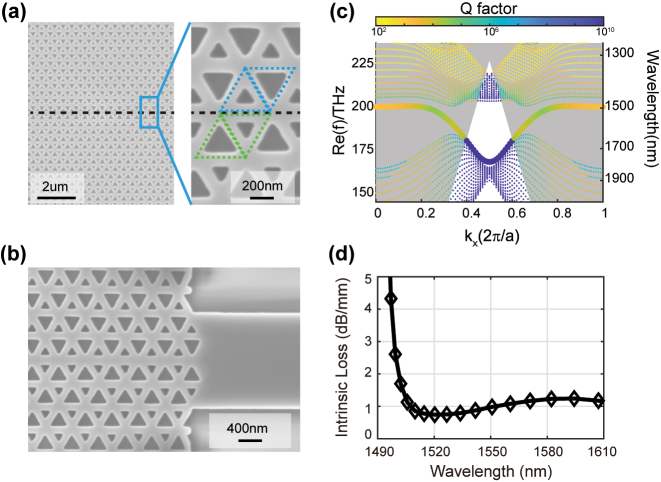
Optical properties of edge states along zigzag-AA topological interface. (a) Scanning electron microscope (SEM) images of zigzag-AA interface. The insets magnify the details of fabricated samples, whose unit cells consist of two inequivalent air holes. The upper and lower domains are constructed by VPC1 (blue) and VPC2 (green). (b) SEM at the facet between the strip waveguide and topological interface. The width of strip waveguide is chosen to be $2\sqrt{3}a$ in order to improve the coupling efficiency. (c) Dispersion of the TE-like projection bands and valley-dependent edge states for zigzag-AA interface. The markers of edge states are enlarged to highlight its dispersion. The color represents the Q factor of photonic crystal (PC) slab modes and grey region is the light cone of silica. (d) Calculated propagation loss of the edge states from the complex-eigenfrequency dispersion. The propagation loss in dB unit can be obtained from $\alpha_{p}=4.34\;*\;2\omega_{i}/\left|v_{g}\right|$ , where *v*_*g*_ = *dω*_*r*_/*dk* is the group velocity of the edge state and *ω*_*i*_ (*ω*_*r*_) is the imaginary (real) part of the frequency.

The boundary of two AA-type interfaces is stacked by two smaller holes, so that they have larger separation between two holes than BB type, which is more accessible in fabrication. On the other hand, the bearded-AA interface has two edge states inside the bandgap, where one is topological and another is trivial. It adds complexity in the analysis of transmittances. Therefore, we chose the zigzag-AA interface to experimentally demonstrate the measurement of loss. To determine the bending and propagation losses in experiment, we should have series of topological interfaces with different turn number or different transmission distance. It requires large-area samples with footprint larger than 375 μm × 265 μm, which contains 414 × 180 air holes with triangular geometry. During the process of electron beam lithography (EBL), we choose subfield compaction mode with beam step size ranging 1 nm to several hundred nm, and finally we optimize the beam step size as 5 nm to balance the exposure time and the acute angle of the triangular holes. Figure 2(a) shows the scanning electron microscope (SEM) images of the fabricated sample along zigzag-AA interface. The inset magnifies the details of fabricated interface, whose unit cell consist of two inequivalent air holes in silicon background and are arranged in honeycomb lattice. The upper and lower domains are constructed by VPC1 (blue) and VPC2 (green), respectively. The black dotted lines highlight the channel of interfaces. As shown in Figure 2(b), both the input and output facets of topological interface are connected with a strip waveguide, while the width of waveguide is optimized to be $2\sqrt{3}a$ for high-efficient coupling.

Subsequently, we should theoretically study the optical properties of valley-dependent edge states for zigzag-AA interface before experiment. In a PC slab, the edge states above the light cone will perform as quasi-guided modes, and thus we should consider the complex-value eigenfrequencies of the edge states to discuss propagation loss. To do this, the guided-mode-expansion (GME) method is applied to compute both the quasi-guided modes above light cone and guided modes below light cone [43]. Figure 2(c) gives the calculated complex-frequency dispersion of the TE-like projection bands and valley-dependent edge states for zigzag-AA interface, where the markers of edge states are enlarged to highlight its dispersion. The color represents the Q factor of photonic crystal (PC) slab modes and grey region is the light cone of silica. Note that most of the edge states are operated above the light cone, and thus those modes will radiate to the out-of-plane direction and manifest intrinsic propagation loss. Such loss in dB unit can be obtained from $\alpha_{p}=4.34*2\omega_{i}/\left|v_{g}\right|$ [45], where *v*_*g*_ = *dω*_*r*_/*dk* is the group velocity of the edge state and *ω*_*i*_ (*ω*_*r*_) is the imaginary (real) part of the frequency. Derived from the complex-frequency edge dispersion in Figure 2(c), we calculate the intrinsic loss of the edge states, as shown in Figure 2(d). The propagation losses are around 0.5–2 dB/mm for calculation in the wavelength intervals from 1496 nm to 1610 nm.

### 2.3 Experimental measurement of bending loss in the topological interfaces

In this and next sections, we will concentrate on the experimental transmittances of zigzag-AA topological interface to evaluate the bending loss *β*_*B*_ and propagation loss *β*_*P*_. Consider a VPC interface integrated on a photonic chip, its transmittance can be modelled as

(3) $$T=T_{0}-\beta_{B}N_{t}-\beta_{P}L,$$

where *T* and *T*_0_ are in dB scale. *T*_0_ is the substrate transmittance including the insertion losses between fibers and grating couplers and between strip waveguides and VPC interfaces, but regardless of bending and propagation losses. *N*_*t*_ is the turn number of 120-deg bending and *L* is the transmission distance inside the interface. According to Eq. (3), we can apply the cut-back method for measurement of bending loss or propagation loss, which is based on a comparison of transmittances through interfaces of different turn number *N*_*t*_ or different transmission distance *L*. Note that there is precondition assuming identical coupling efficiency and identical side wall roughness of the samples.

**Figure 3:**
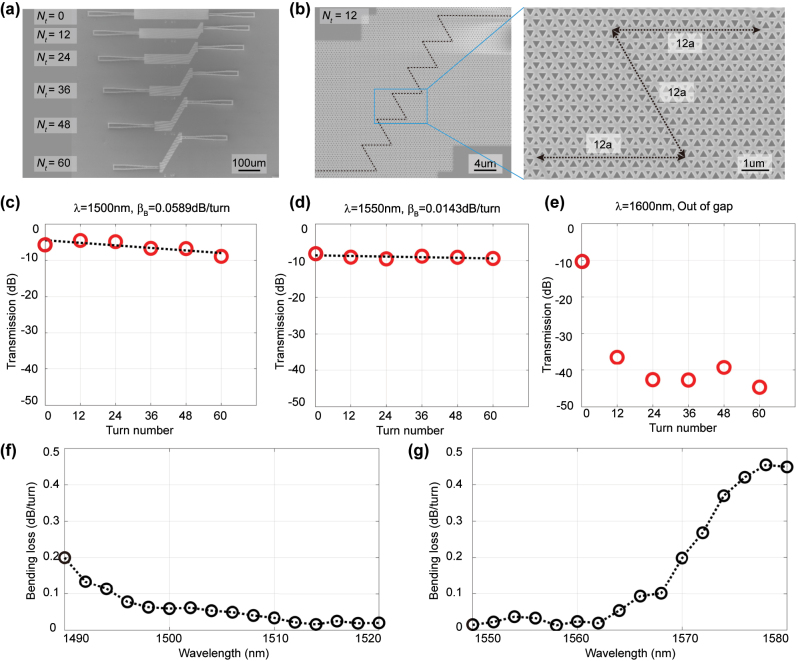
Experimental measurement of bending loss in the topological interfaces with different turn number. (a) Overall view of scanning electron microscope (SEM) image for the fabrication sample. Here is one set of the samples that contain six types of topological interfaces with different turn number, ranging from 0 to 60. These interfaces have a fixed transmission distance $L_{0}^{\prime }$ = 768*a*. (b) SEM image of the topological interface with *N*_*t*_ = 12. The inset magnifies the detail of a Z-shape bending. (c–e) Transmittances as a function of turn number at three fixed wavelengths of 1500 nm, 1550 nm and 1600 nm. The data of red dots are averaged by the transmittances of five duplicated samples in an individual chip. The dash black lines are the linear fitting of the measured data, and the slopes of these lines indicate that the bend losses of measurement are 0.0589 dB/turn for *λ* = 1500 nm, and 0.0143 dB/turn for *λ* = 1550 nm. (f–g) Bending loss as a function of wavelength at two wavelength regimes. Inside the photonic bandgap from ∼1490 nm to ∼1570 nm, the measured bending losses are less than 0.2 dB/turn.

To determine the bending loss, we should fix the transmission distance of interface as a constant $L_{0}^{\prime }$ , and then obtain a set of samples with different turn number *N*_*t*_. In this way, Eq. (3) can be rewritten as,

(4) $$T=T_{0}^{\prime }-\beta_{B}N_{t},$$

where $T_{0}^{\prime }=T_{0}-\beta_{P}L_{0}^{\prime }$ is a fixed substrate transmittance even for different *N*_*t*_. The transmittances of topological interfaces will reduce by increasing the turn number, and thus the reduction of transmittance per turn can be fitted. To do this, we fabricated one set of the samples that contain six types of topological interfaces as the SEM image shown in Figure 3(a), and duplicated 5 sets of the samples in an individual chip. The turn number of one set of samples range from 0 to 60, while the interfaces have a fixed transmission distance $L_{0}^{\prime }$ = 768*a* and assume maintaining the same substrate transmittance $T_{0}^{\prime }$ . Taking the case of *N*_*t*_ = 12 as an example, Figure 3(b) shows the corresponding SEM image with six Z-bending units. The inset magnifies the detail of a Z-shape bending, comprising three 12-*a*-length edges highlighted by the black dashed lines. Note that the length of edges at sharp bending corners keep as 12*a* for all samples with different *N*_*t*_.

In experiment, the transmittances of topological interfaces are measured based on a fiber-to-waveguide vertically coupled system [see Section 4.2 the experimental measurement for more details]. Based on the above optical setup, we can obtain the transmission data of samples that contains 6 configurations with different turn number. As shown in Figure 3(c–e), the transmittances as a function of turn number *N*_*t*_ are plotted at the three fixed wavelengths of 1500 nm, 1550 nm, and 1600 nm, respectively. All of transmittances of topological interfaces are normalized by the transmittances through the $2\sqrt{3}a$ -width strip waveguides, and are averaged by the independently measured data of duplicated 5 sets of samples. According to Eq. (4), we have a linear fitting for the transmittance data and the slopes of these lines indicate the bending losses of measurement *β*_*B*_. We have obtained ultralow bending losses, i.e. 0.0589 dB/turn for *λ* = 1500 nm and 0.0143 dB/turn for *λ* = 1550 nm. Note that, due to the deviation of fabrication dimensions of VPC interfaces, the measured results indicated the operation wavelengths had a blue shift and the photonic bandgap changed to be from ∼1490 nm to ∼1570 nm. Therefore, the transmittance decreased rapidly from flat interface (*N*_*t*_ = 0) to sharp-bend interface (*N*_*t*_ ≠ 0) at *λ* = 1600 nm, because this wavelength operates inside the bulk states of the first TE-like band. One advantage of VPC interfaces is the broadband operation of robustness against sharp bend. To manifest this feature, Figure 3(f) and (g) show the bending loss spectrum as a function of wavelength at two wavelength regimes. Inside the photonic bandgap from ∼1490 nm to ∼1570 nm, the measured bending losses are less than 0.2 dB/turn. Note that there is a regime (from 1520 nm to 1550 nm) that don’t show the results, because the strong confinement of the edge modes in this regime makes the propagation efficiency of bending interface be larger than that of flat interface at some wavelengths, and the estimation of bending losses are tricky to be negative values. Nevertheless, regardless of such tricky regime, the results in Figure 3(f) and (g) are enough to justify the broadband feature of ultralow bending loss in VPC topological interfaces.

To highlight the ultralow bending loss in this work, we further compare our results to the measured bending loss in some other waveguides of PICs, as shown in Table 1. Conventionally, the rectangle waveguides in PICs focus on achieving 90-deg bending [8], [9] and the loss is reduced to be 0.06 dB/turn by controlling the skin depth of evanescent waves [7]. For more sharp bending (e.g. 120°), the mechanism of photonic bandgap is introduced by precisely tuning the building blocks around corner [11], but the measured bending loss is larger than others because the performance will dramatically suffer from fabrication imperfections. Our method based on topological protection has achieved the lowest bending loss among these methods, and this feature has less effect on fabrication imperfections. Through these results, we have experimentally demonstrated VPC with ultra-low bending losses, which is expected to achieve compact-size photonic devices with dense integration and extend the information processing capabilities of modern single-chip devices.

**Table 1:** Bending loss comparison between valley-Hall topological method and conventional methods.

Method	Bending loss	Bent angle	Wavelength	Mechanism
Strip waveguides [8]	∼ 0.5 dB/turn	90^o^	1550 nm	TIR at a certain radius
Rib waveguides [9]	0.32 dB/turn	90^o^	1550 nm	TIR with a reflector at the corner
Extreme skin-depth waveguides [7]	0.06 dB/turn	90^o^	1550 nm	Photonic skin-depth engineering of evanescent waves
Line-defect photonic crystals [11]	∼1 dB/turn	120^o^	∼1400 nm	Corner modification in photonic bandgap
This work (valley photonic crystals)	0.0143 dB/turn	120^o^	1550 nm	Topological protection

TIR, total internal reflection.

### 2.4 Experimental measurement of propagation loss in the topological interfaces

**Figure 4:**
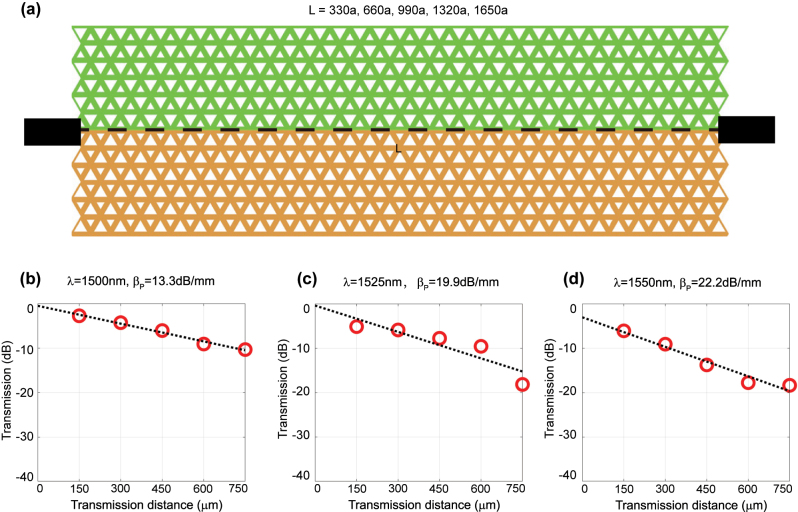
Experimental measurement of propagation loss in the topological interfaces with different transmission distance. (a) Schematic view of the topological interfaces for transmission-loss measurement. *L* is the transmission distance of interfaces ranging from 330*a* to 1650*a*, in order to estimate the propagation loss. (b–d) Transmittances as a function of transmission distance at three fixed wavelengths of 1500 nm, 1525 nm, and 1550 nm. The data of red dots are averaged by the transmittances of five duplicated samples in an individual chip. The black dashed line is the data line after linear fitting, and the slope of the line represents the measured propagation loss. The propagation losses of measurement are larger than 10 dB/mm.

In this section, we will measure another key parameter of topological interfaces, i.e. propagation loss *β*_*P*_. Based on the cut-back method, we fix the turn number as a constant $N_{t}^{\prime \prime }$ , and then obtain a set of samples with different transmission distance *L*. In this way, Eq. (3) can be rewritten as,

(5) $$T=T_{0}^{\prime \prime }-\beta_{P}L,$$

where $T_{0}^{\prime \prime }=T_{0}-\beta_{B}N_{t}^{\prime \prime }$ is a fixed substrate transmittance even for different *L*. The transmittances of topological interfaces will reduce by increasing the transmission distance *L*, and thus the reduction of transmittance per millimeter can be fitted (i.e. propagation loss in the unit of dB/mm). Figure 4(a) give the schematic view of the topological interfaces for propagation-loss measurement, where *L* is the transmission length of interfaces ranging from 330*a* to 1650*a*. Then we test the transmittances of these samples prepared by different transmission distances. To estimate the propagation loss, the curves of transmittances are plotted as a function of transmission distance at different wavelengths, as shown in Figure 4(b–d). The lines are the linear fitting of the measured data, and the slopes of these lines indicate that the propagation loss of measurement, i.e. 13.3 dB/mm for 1500 nm, 19.9 dB/mm for 1525 nm, and 22.2 dB/mm for 1550 nm, respectively. In the asymmetric slab of our VPC interfaces, such measurement of propagation loss is larger than 10 dB/mm primarily due to (i) the intrinsic out-of-plane radiation above light cone and (ii) the extrinsic out-of-plane radiation caused by the sidewall roughness. The loss can be reduced by an order of magnitude in principle, if we improve the fabrication techniques to decrease the sidewall roughness as well as achieve the interface in a free-standing slab, i.e. air-PC-air sandwiched structure. Nevertheless, propagation loss is an inevitable issue for photonic crystal slab, and thus VPC is more suitable for the design of compact-size devices, instead of long-distance communication applications.

## 3 Conclusions

In summary, we have characterized the robustness of sharp-bending integrated topological interfaces based on VPCs and qualified the ultralow bending loss. By breaking the inversion symmetry of honeycomb lattice, the VPCs have been designed on 220-nm-thickness silicon layers, which are asymmetrically cladded by the top air region and the bottom silica substrate. We have shown four types of valley-dependent topological interfaces, and employed the representative case of zigzag-AA interface to demonstrate the bending and propagation losses. The measured results have experimentally verified the bending loss in the zigzag-AA interface less than 0.02 dB/turn. This work gives a fundamental understanding on the experimental exploration of sharp-bending robustness protected by valley topology. Similarly, the method we used to experimentally quantify bending loss is accessible for other types of VPC interfaces and other types of topological phases in PICs. Based on the fact of ultra-low bending loss in this work, the VPCs will perform superiority on the design of sharp-bending devices, which is promising for densely integrated photonic chip.

## 4 Methods

### 4.1 Sample fabrication

The samples in this work were manufactured by employing a top-down nanofabrication process on a SOI wafer (with a nominal 220-nm-thick device layer and a 2-μm-thick buried oxide layer). First, a 400-nm-thickness positive resist (ARP6200) was spun with a rotating speed of 4000 min^−1^ on the wafer and dried for 10 min at 180 °C, where the resist act as a hard mask for subsequent mask. To precisely define the patterns of VPC structures, the resist was exposed by electron-beam lithography (EBPG5000+, Riath), and developed by dimethylbenzene for 70 s. Note that we chose Subfield Compaction mode and the beam step size was set as 5 nm. Second, inductively coupled plasma (ICP) etching step was applied to etch the VPC structures, strip waveguides and grating couplers on the top 220-nm-thickness silicon layer. Finally, the resist was removed through oxygenating the etched structure and the device layer appeared on the top of the chip.

### 4.2 Experimental measurement

The optical measurement in this work is based on a vertically coupled system. For light source, we used wavelength-tunable continuous-wave lasers (Santec TSL-710), which can be tested in the wavelength range from 1480 nm to 1640 nm. Emitted from the laser, the CW light at a certain wavelength firstly launched on a fiber polarizer to select TE polarization. Then the signal in fiber is vertically coupled to one of the grating couplers and converted into the in-plane propagating waves along the strip waveguides and the topological interfaces. Similarly, the output signal after passing through the interfaces is collected by another fiber and is measured by the optical power meter (Santec MPM-210).
